# Recent Advances in Bioactive Compounds, Health Functions, and Utilization of Rose (*Rosa* spp.)

**DOI:** 10.3390/molecules30193869

**Published:** 2025-09-24

**Authors:** Xinxin Zhao, Yuqin Jiang, Mingfeng Qiao, Fangjun Lin, Baohe Miao

**Affiliations:** 1 Institute of Urban Agriculture, Chinese Academy of Agricultural Sciences, Chengdu National Agricultural Science & Technology Center, Chengdu 610213, China; zhaoxinxin@caas.cn; 2 Department of Food Science & Technology, School of Agriculture & Biology, Shanghai Jiao Tong University, Shanghai 200240, China; yuqinj07@sjtu.edu.cn; 3 Culinary Science Key Laboratory of Sichuan Province, Sichuan Tourism University, Chengdu 610100, China; mfqiao@163.com; 4 Department of Biotechnology, College of Animal & Veterinary Science, Southwest Minzu University, Chengdu 610041, China

**Keywords:** rose, bioactive compounds, bioactivities, utilization, by-products

## Abstract

Our review systematically outlines the bioactivity and industrial applications of key functional compounds in roses, with a particular focus on their potential in food and pharmaceutical industries from technical, economic, and commercialization perspectives. We summarize the evidence supporting the efficacy of rose-derived polyphenols, flavonoids, and essential oils in areas including antioxidant, anti-inflammatory, antibacterial, and neuroprotective effects, which provide a scientific basis for their use in functional foods and preventive medicines. We further evaluated the commercial viability of processing rose by-products. Additionally, we analyze current and potential applications of rose bioactive compounds in natural food preservatives, functional dietary supplements, herbal medicines, and cosmetic products. Finally, we discuss the remaining challenges and future directions for the industrial utilization of roses, including standardization, efficacy validation in humans, and scalable economic models, to facilitate the transition from experimental research to commercially sustainable applications.

## 1. Introduction

Roses (*Rosa* spp.) are the most romantic and widely used ornamental plant worldwide. They have long been valued for their broad applications in landscaping, ecological conservation, perfumery, cosmetics, food processing, as well as medical and health care [1,2]. In recent years, there has been growing scientific and commercial interest in roses due to their rich profile of bioactive compounds, including essential oils, polyphenols, flavonoids, polysaccharides, and vitamins [3,4], which are associated with a wide range of health benefits such as antioxidant, anti-inflammatory, antimicrobial, and antidiabetic activities, as well as protective effects on different tissues [5,6].

*Rosa* spp., a genus within the family Rosaceae, comprise approximately 317 species and thousands of cultivated varieties. Many of these were previously world-famous ornamental plants without biological applications. They are rich in natural antioxidants and functionally active compounds, which contribute to significant nutritional and bioactivities [1]. This review focuses on five species that are most widely used in industrial applications: *Rosa damascena* Mill., *Rosa rugosa* Thunb., *Rosa canina* L., *Rosa roxburghii* Tratt., and *R. laevigata* Michx. We elaborate on the major bioactive compounds derived mainly from their petals and fruits. Moreover, the potential for the industrial exploitation of roses, particularly in functional foods and pharmaceuticals, remains underexplored, and large quantities of rose by-products generated during essential oil and floral water extraction are often discarded, leading to resource waste and environmental concerns. Therefore, this review presents new findings on the bioactive compounds and health benefits of roses and their by-products.

Herein, we reviewed high-quality literature from the Web of Science, PubMed, and Scopus published in recent years. This review summarizes the advances in the principal bioactive compounds and key biological functions of *Rosa* species, with a particular focus on rose bioactive ingredients and their practical, economically viable applications in industry. Ultimately, this study seeks to provide a roadmap for the integrated and sustainable utilization of rose resources, supporting the transition from laboratory evidence to industrial applications.

## 2. Bioactive Compounds in *Rosa* spp.

Rose species are rich in a diverse array of bioactive compounds, which contribute significantly to their nutritional, medicinal, and functional properties. The major classes of bioactive constituents include phenolic compounds, polysaccharides, vitamins, carotenoids, and essential oils [7,8]. These phytochemicals are primarily concentrated in the petals and fruits, which represent the most studied and commercially utilized parts, driving applications in functional foods, nutraceuticals, cosmetics, and pharmaceuticals.

**Figure 1:**
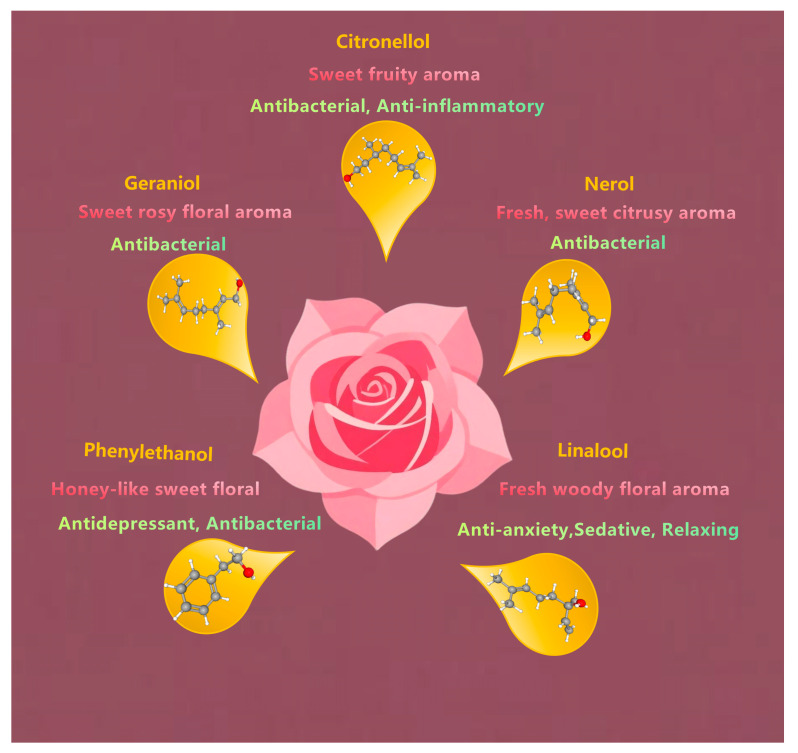
The main components and effects of rose essential oil.

Rose essential oil is a valuable aromatic oil extracted from roses (Figure 1); its composition, however, varies considerably across different rose species [9]. Geraniol (42.08%), heneicosane (22.07%), n-heptadecane (16.70%), and linalool (11.55%) are the main constituents of the essential oil from *R. canina* flowers [10,11]. The total monoterpenoid content of *R. rugosa* essential oil is 70.31%, of which the linalool content is 33.73% [6]. In addition, the relative contents of geraniol and citral are relatively high in the four types of rose essential oils [12]. GC-MS analysis identified citronellol and geraniol as the predominant constituents within the essential oil composition, although their relative abundances exhibited significant quantitative fluctuations in response to variations in temperature and precipitation levels [13].

In the past decade, there has been a new research focus in the rose industry on the recovery and utilization of by-products after the extraction of rose essential oils. Rose waste has potential applications in food production owing to its rich in bioactive substances, including flavonoids, anthocyanins, polysaccharides, and dietary fiber [14,15]. It not only extends the industrial chain and enhances industrial value, but also facilitates the recycling of waste materials and reduces environmental impact.

In *R. damascena* residue water, the main compound is phenylethyl alcohol, which is also rich in polyphenols, including epicatechin and hesperidin. This indicates that the composition of polyphenols in *R. damascena* wastewater is similar to that of rose essential oils [16], with quercetin and kaempferol glycosides being the major polyphenol compounds. After freeze-drying, *R. rugosa* residue also contains high levels of polyphenols and anthocyanins [17]. Additionally, polysaccharides are essential components of rose residues, especially water-soluble pectic extracts of *R. damascena* residues [18]. Two pectic polysaccharide components, WSRP-2A and WSRP-2B, have been identified in *R. setate* × *R. rugosa* waste [19]. However, the soluble dietary fiber content of rose residue is relatively low, and most dietary fiber is insoluble. Thus, improving the utilization of this dietary fiber remains a problem to be addressed [20].

## 3. Bioactivities

The bioactive compounds in roses demonstrate a wide range of biological effects, such as antioxidant, anti-inflammatory, antibacterial, anti-diabetic, anti-cancer activities, as well as protective effects on the nervous system, gastrointestinal tract, liver, and skin (Table 1).

### 3.1. Antioxidant Effect

The antioxidant activity of roses mainly comes from essential oil and extracts. Most studies have focused on the antioxidant activities of rose petals. The antioxidant activity of rose essential oils mainly arises due to terpene compounds, especially citronellol, geraniol, nerol, linalool, and phenylethanol. Meanwhile, the extracts of rose (such as water extracts, ethanol extracts, and supercritical CO_2_ extracts) are rich in phenolic compounds, such as flavonoids (quercetin, kaempferol glycoside), phenolic acids (gallic acid), and tannic acid and its derivatives.

Studies show that the phenol contents are positively correlated with antioxidant capacity, with phytochemicals being most prominent in unfolded petals [43]. It has also been reported that antioxidant activity is highly correlated with the total phenolic content, but it is not affected by the harvest seasons of the *R. rugosa* petals [44]. Moreover, two new isoflavones isolated from the flowers of *R. damascena* have been shown to exhibit good antioxidant activity with IC_50_ values of 4.2 and 4.0 μg/mL [45]. However, there are five different Rosa extracts that effectively reduced malondialdehyde levels and enhanced the activities of key antioxidant enzymes, superoxide dismutase, and glutathione peroxidase in the H_2_O_2_-induced HaCaT cell model, highlighting their significant potential for development as novel antioxidant agents [46]. Research indicates that antioxidant-rich *R. damascena* significantly reduces oxidative stress in high-risk pregnant women, supporting its potential as an herbal-based preventive intervention for the management of high-risk pregnancies [22]. A study demonstrated that *R. rugosa* extract exhibits significant, dose-dependent antioxidant activity, scavenging up to 82% of free radicals at 50 mg/mL in vitro [47]. Studies in Nrf2-knockout zebrafish model demonstrated that *R. rugosa* extracts confers oxidative stress resistance and upregulates antioxidant gene expression (gstp1, prdx1) through an Nrf2-dependent mechanism [21]. Further analysis revealed that, in human endothelial cells, the polyphenolic composition of *R. canina* fruits significantly increased the levels of the antioxidant glutathione molecule while decreasing reactive oxygen species [48].

Several studies have analyzed the antioxidant effects of polysaccharides. One study focused on polysaccharides from *R. rugosa* petals (RRPS) and determined their in vitro antioxidant activities. This study suggests that RRPS-2 has good potential for scavenging radicals [49]. Antioxidant activity assays revealed that *R. roxburghii* polysaccharides possess significant free radical scavenging capabilities, with IC_50_ values of 0.45 mg/mL and 0.53 mg/mL against DPPH and ABTS radicals, respectively [46]. Furthermore, a novel water-soluble polysaccharide (RRTP1-1) has been obtained from *R. roxburghii* fruit using ultrasonic-assisted extraction. In vitro antioxidant tests have shown that RRTP1-1 exhibits significant scavenging activity against DPPH, hydroxyl, and superoxide radicals. Moreover, in vivo antioxidant assays have shown that RRTP1-1 at 200 or 400 mg/kg significantly enhances the activities of antioxidant enzymes, increases total antioxidant capacity values, and decreases lipid peroxidation level and Malonaldehyde (MDA) levels to different degrees in the serum of D-Gal aging-induced mice [50].

### 3.2. Anti-Inflammatory Activity

Inflammation is a basic pathogenic factor involved in tissue injury and pain as well as in acute and chronic diseases. Previously, the structural types of anti-inflammatory natural products discovered from plants mainly included terpenoids, flavonoids, alkaloids, and polysaccharides. Roses are known as medicinal plants in Asia and have protective effects against inflammation-related diseases that have been widely reported, including lung inflammation [51], allergic inflammation [24], skin inflammation [52], and other immunomodulatory activities [53]. Rugosic acid A, extracted from *R. rugosa* petals, has been shown to exhibit anti-inflammatory effects in LPS-mediated RAW 264.7 cells and a lung injury model. It can disrupt IL-6-mediated STAT3 activation and ameliorate LPS-induced nitric oxide (NO) production and nuclear translocation of NF-κB [51]. Epidermal-growth-factor-induced A549 cells have been used to investigate inflammation in allergic asthma and extracts from *R. laevigata* have been found to inhibit NF-kappa B activity and COX-2 expression [24]. JB6 P+ cells have been used to evaluate the anti-inflammatory activity of the skin and the results demonstrated that rose petal extract (RPE) reduced both cytokine production and expression of SUV-induced COX-2 [52]. In addition, *Rosa* extracts also reduce mouse thymus inflammation [25]. The anti-inflammatory effect of the extract was dose dependent, as it inhibited NO production in LPS-stimulated RAW 264.7 macrophages [54]. Because severe adverse effects are caused by the long-term use of synthetic steroids and non-steroidal anti-inflammatory drugs, novel effective materials with minimal side effects are required [26]. RT50 could reduce the pro-inflammatory cytokines and chemokines stimulated by tumor necrosis factor-α/interferon-γ in HaCaT cells and lower the elevated levels of Immunoglobulin E/Immunoglobulin G caused by DNCB exposure, demonstrating promising therapeutic potential for inflammatory skin conditions by upregulating key skin barrier proteins (filaggrin, involucrin, and loricrin) [23]. RLPa-2 inhibited the production of inflammatory factors (NO, IL-6, and TNF-α), and the expression of pro-inflammatory proteins (p-STAT3/STAT3) in LPS and IFN-γ-induced M1 macrophage [55]. The anti-inflammatory mechanism of roses is mainly related to their various bioactive components. These components exert anti-inflammatory effects by regulating inflammatory signaling pathways, inhibiting the release of pro-inflammatory factors, and scavenging free radicals. Therefore, *Rosa* could be a natural biomaterial resource for the development of safe and effective pharmacological immunomodulatory agents.

### 3.3. Antibacterial Activity

Treating infections caused by antibiotic-resistant bacteria is challenging, and researchers are looking for new antimicrobial compounds. The antibacterial activity of rose essential oil has mainly been determined according to the total content of phenylethyl alcohol, citronellol, geraniol, neroli, linalool, and eugenol [56], while *Rosa* extracts and their natural products, such as phenols, linalool, phenylethyl alcohol, citronellol, and bisabol, show a broad spectrum of antibacterial activity [57]. This makes it a valuable ingredient in natural skincare, food preservation, and pharmaceutical products.

Different extraction approaches, such as ethyl acetate [58,59], ethanol [57], and aqueous [60] extraction, have been studied to evaluate their influence on antimicrobial activity. The antibacterial effects of different extracts of *R. canina* have been examined against pathogenic *Gram-negative bacilli* [4]. The ethyl acetate extract from the petals of *R. damascena* has shown in vitro anti-plasmodial activity [59]. The aqueous extract of *R. rugosa* fruit exhibited activity against over 10 bacteria, including *Bacillus cereus*, *Escherichia coli*, *Klebsiella pneumoniae*, *Enterococcus faecalis*, *Proteus mirabilis*, *Pseudomonas aeruginosa*, *Proteus mirabilis*, and *Enterococcus faecalis*. However, bacteria such as *Salmonella enteritidis*, *Staphylococcus aureus*, *Proteus mirabilis*, *Listeria innocua*, *Enterococcus faecalis*, and *Staphylococcus epidermidis* have shown the highest levels of resistance to ethanolic extracts [60].

The antibacterial activity of rose extracts also varies among different parts of the plant. An interesting finding was that, in the antimicrobial evaluation against one fungal and eight bacterial strains, the leaf extracts demonstrated the greatest efficacy, followed by petal and hip extracts, whereas root extracts showed the weakest activity [61]. Due to the unique physical and chemical properties, metal-based nanoparticles have gained special attention for development as antimicrobial nanomaterials. Rosehip-extract-functionalized magnesium-based nanoparticles showed enhanced antibacterial activity against both Gram-positive (*S. epidermis* and *S. aureus*) and Gram-negative (*E. coli*) bacteria, as well as enhanced in vivo efficacy in an invertebrate model infected with *S. aureus* bacteria [27].

Roses and their derived extracts exhibit significant potential for application as natural preservatives within the cosmetic and food industries, as well as formulations for pet care. Their historical and ongoing use in traditional medicine for the management of infections and dermatological conditions underscores their therapeutic relevance. However, the realization of this potential necessitates the targeted selection of specific rose varieties and appropriate extraction methodologies based on the intended target microorganism species. Consequently, rigorous validation of their demonstrated efficacy under relevant conditions is imperative prior to deployment in these applications.

### 3.4. Anti-Diabetic Effect

Diabetes mellitus is a multisystemic and common metabolic disorder characterized by chronic hyperglycemia that may affect the eyes, kidneys, vessels, and heart. Chronic hyperglycemia causes the non-enzymatic glycation of proteins and elevation of the polyol pathway, resulting in oxidative stress that damages organs [62]. The main active components contributing to roses’ anti-diabetic effects include polyphenolic compounds (such as ellagic acid and gallic acid), flavonoids (including quercetin and kaempferol), terpenoids (notably citronellol and geraniol), and polysaccharides.

There has been a surge in research on the use of natural products to manage diabetes [3,63]. Polysaccharides in *R. roxburghii* exhibit strong antioxidant and α-d-glucosidase-inhibitory activities [64]. Selenium nanoparticles functionalized with polysaccharides from *R. roxburghii* fruit have also shown greater protective effects on INS-1 cells against H_2_O_2_-induced cell apoptosis [65]. The flavonoids isolated from *R. roxburghii* have great inhibitory effects on α-glucosidase in vitro, and research has shown that the inhibitory effects of flavonoids decrease after simulated gastric digestion [66]. Water-soluble polysaccharides extracted from *R. roxburghii* exhibit favorable inhibitory activities against α-glucosidase in a mixed-inhibition manner [67]. An in vivo study has indicated that the levels of fasting blood glucose, serum insulin, and serum lipids in type-2 diabetic db/db mice are decreased by the oral administration of a polysaccharide isolated from *R. roxburghii* fruit [28]. Polyphenols extracted from *R. rugosa* Thunb regulate lipid metabolism in diabetic rats by activating the AMPK pathway and increasing the expression of FGF21 in the liver [29]. Another diabetes study conducted on animal models revealed that purified oligosaccharide isolated from *R. canina* can stimulate the regeneration of β-cells in the islands of langerhans, resulting in a significant increase in insulin levels [30].

The anti-diabetic effect of roses is centered on polyphenols and flavonoids, and it is achieved through multiple pathways such as AMPK/PI3K-Akt signaling activation, β-cell protection, inhibition of glycometabolic enzymes, and anti-inflammation. Future research should focus on the mechanism of component synergy and clinical translation to promote its application as an adjuvant therapy.

### 3.5. Anti-Cancer Effect

Currently, tumor monotherapy cannot meet clinical needs, such as high doses, poor efficacy, and the emergence of drug resistance. Combination therapy is a new approach to overcoming these problems [68]. The active components in roses exhibit significant anti-cancer effects and possess considerable potential application as an anti-cancer dietary supplement.

Various polysaccharide-rich extracts of roses have shown in vitro antiproliferative activity against human lung cancer and colon cancer [69]. The extract of *R. canina* induced cell cycle arrest at the G1 phase and triggered apoptosis by reducing mitochondrial membrane potential and increasing caspase activity in A549 and prostate cancer (PC-3) cells [70]. Research shows that *R. rugosa* seed oil significantly inhibited the spontaneous migration of fibroblasts and A549 cells and reduced the formation of reactive oxygen species, with a higher emulsion activity in A549 cultures [71]. Polysaccharide-rich extracts from petals of *R. rugosa* have been studied for their composition and influence on various cellular processes involved in the development of cancer and other civilization diseases [69].

In vivo experiments showed that, as the concentration of the *R. laevigata* fruits-derived polysaccharide (JYP70–1) increased, the expression levels of phosphorylated FAK (Tyr397) and matrix metalloproteinase-2 decreased, which indicates that JYP70-1 regulates the FAK signaling pathway to inhibit the migration of HepG2 cells [31]. The anti-cancer effect and the underlying mechanism of action of the ethanolic extract of *R. cymosa* fruits was investigated both in vitro and in a xenograft animal model. *R. cymosa* fruits’ ethanolic extracts significantly reduced the tumor size by activating phosphatase and tensin homolog and impairing the PI3K/Akt/Foxo and Jak/Stat activation pathways [72]. When treated with an herbal combination, including *R. rugosa*, *Cyperus rotundus* L., and *Citrus medica* L., the tumor weight and volume, and the level of estradiol in serum were reduced in a breast cancer rat model, and the protein expression of SNCG, ER-α, p-AKT, and p-ERK was decreased in breast cancer (MCF-7 and T47D) cells [32]. A randomized controlled clinical trial reported that different concentrations of *R. damascena* essential oil improved the sleep quality of patients with cancer [73].

The anti-cancer mechanisms of roses are characterized by a dual-core system: the polyphenol/flavonoid-mediated induction of apoptosis and cell cycle arrest, coupled with polysaccharide-driven immunomodulation. This core activity is augmented by multidimensional effects, including the inhibition of metastasis and angiogenesis. Overcoming bioavailability limitations and advancing clinical validation remain critical for future translational research.

### 3.6. Neuroprotection

Human life expectancy increases as living conditions improve significantly, and an increasing number of people are suffering from neurodegenerative diseases such as Parkinson’s, Alzheimer’s, and senile dementia [74]. New approaches are needed to treat these pathologies because most neurodegenerative disease clinical trials do not obtain efficacious results [75].

Researchers have investigated the neuroprotective effect of selenium-containing polysaccharides from the fruit of *R. laevigata* against oxidative stress induced by H_2_O_2_ in SH-SY5Y neuroblastoma cells, finding that Se-RLFP-1 exhibited obvious neuroprotective activity at a concentration of 100 μg/mL [76]. Two pure polyhydroxy triterpenoids isolated from *R. laevigata* fruit have shown high acetylcholinesterase (AChE) inhibitory activities, with IC_50_ values of 29.22 and 45.47 μg/mL, as well as exhibiting potential neuroprotective activities against H_2_O_2_-induced SHSY5Y cell death [76]. Researchs have also evaluated five cultivars of *R. damascena*, all of which had high potency for scavenging free radicals and inhibiting acetylcholinesterase activity, with a high IC_50_ value of 3.92 μg/mL [77]. According to the above findings, the compound in *Rosa* extracts can inhibit the activity of acetylcholinesterase and may have the potential to be used as a drug to prevent and treat senile dementia.

Aaquatic *R. damascena* extract has a protective effect against the oxidative damage induced by AlCl_3_ intoxication in an Alzheimer’s model [33]. This is because the *R. damascena* extract increased catalase and glutathione levels, attenuated MDA levels, and regulated acetylcholinesterase activity. A clinical trial assay evaluated the effects of aromatherapy with *R. damascena* on pain and anxiety reduction during the first stage of labor and revealed that pain severity and anxiety levels were significantly lower in the treatment group [34]. An ultrasonic nebulizer was used to apply aromatherapy to patients for 15 min before they went to the operating room for surgery. The treatment group showed a significant difference from the control group in the second STAI-S test [35].

The neuroprotective effects of rose, particularly its petals and rosehips, result from multi-target and multi-pathway synergistic actions. The core mechanisms lie in its exceptional antioxidant and anti-inflammatory capacities, which directly counteract the two primary contributors to neuronal damage, including oxidative stress and neuroinflammation. Furthermore, by activating endogenous protective pathways, safeguarding critical cellular organelles, inhibiting detrimental enzyme activities, and potentially modulating neurotransmitter systems, rose constituents provide a more comprehensive protective barrier for neurons. These mechanisms underpin its potential therapeutic applications in the prevention and adjunctive treatment of neurodegenerative diseases, cerebral ischemic injury, and neuroinflammatory disorders, as well as in the improvement of cognitive function.

### 3.7. Gastrointestinal Protection

Lifestyle and diet affect the occurrence and development of gastrointestinal diseases [78]. Polysaccharides from roses have been found to exhibit excellent gastrointestinal protective properties.

One study investigated the digestibility and prebiotic potential of a novel polysaccharide from *R. roxburghii* fruit using an in vitro fermentation model and found it improved some beneficial gut microbiota via modulating the microbial structure, lowering the ratio of Firmicutes to Bacteroidetes from 14.89 to 4.68 after 48 h of fermentation [79]. Furthermore, a fecal microbiota transplantation study was conducted to demonstrate that polysaccharides from *R. roxburghii* fruit exhibited prebiotic effects on mice with high-fat-diet-induced colitis. The fecal microbiota of *R. roxburghii* fruit-polysaccharide-treated donor mice significantly alleviated body weight loss, gut microbiota dysbiosis, loss of barrier integrity, and colonic inflammation, while upregulating the expression of tight junction proteins [37]. Oral supplementation of *R. laevigata* polysaccharides ameliorated the typical symptoms of ulcerative colitis, such as body weight loss and diarrhea, by increasing the abundance of beneficial bacteria and reducing the abundance of harmful bacteria [36]. The contractile responses of the intestinal smooth muscles are due to periodic depolarization and repolarization. The juice mixture of *R. canina* had a protective effect against indomethacin-induced gastric lesions and antagonized the effects of indomethacin on apoptosis and lipid peroxidation in a rat mode [80].

The gastrointestinal protective effects of rose center on reinforcement of the mucosal barrier and dual-pathway inhibition of inflammation and oxidative stress. By inhibiting gastric acid secretion, promoting tissue repair, modulating gut microbiota, and regulating smooth muscle function, rose acts multi-dimensionally to maintain gastrointestinal homeostasis. Its natural multi-component synergistic properties confer significant development potential for functional gastrointestinal disorders and mucosal injury repair, although further standardized research is necessary to validate clinical applications.

### 3.8. Hepatorenal Protection

Recent evidence highlights the efficacy of rose polyphenols and polysaccharides in mitigating diabetic nephropathy, cisplatin nephrotoxicity, non-alcoholic fatty liver disease, and drug-induced liver injury.

*R. roxburghii* fruit is rich in multiple bioactive compounds with hepatorenal protective activities. It has previously been shown to possess antifibrotic properties in chronic renal diseases. Freeze-dried *Rosa* fruit powder efficiently alleviated pathological changes in the kidneys of unilateral ureteral obstruction rats by inhibiting oxidative stress and TGF-β1/Smads signaling [38]. *R*. *laevigata* polysaccharide modulated tryptophan metabolism, inhibited ferroptosis in the kidneys, and prevented apoptosis mediated by the PI3K/AKT pathway, showing significant therapeutic effects on diabetic nephropathy mice [39].

Total flavonoids from *R. laevigata* fruit exhibited protective activity against lipopolysaccharide-induced liver injury [40]. *R. Laevigata* extract intragastrically administered for seven days improved liver pathological changes by altering farnesoid X receptor-mediated oxidative stress, inflammation, and lipid metabolism in the liver injury mice models [40].

The hepatorenal protective effects of rose are primarily mediated through Nrf2-driven antioxidant defense, suppression of NF-κB/NLRP3-dependent inflammation, and inhibition of TGF-β-induced fibrotic signaling. However, clinical translation requires enhanced bioavailability of active constituents and validation in human trials.

### 3.9. Skin Protection

Current evidence shows that different extracts from *Rosa* are used in skin protection as antioxidation [56], anti-aging [81,82], whitening [83], moisturizing, anti-acne [84], anti-allergy, and sunscreen agents [56], inhibiting all kinds of melanin enzymes [57], blocking the melanocyte signaling pathway [52], inhibiting melanocyte proliferation, and reducing ultraviolet radiation [85].

Research found that the flower cell sap of *R. rugosa* exhibited strong tyrosinase inhibitory activities [56]. Kojic acid has also been detected in ethanol extracts of *R. damascena* as a tyrosinase inhibitor [57]. In vitro studies showed that the RRPS significantly reduced nitric oxide (NO) production on RAW264.7 macrophages, indicating anti-inflammatory effects [42]. Furthermore, ultrasonic extracts from *R. canina* L. are excellent sources of natural anti-tyrosinase inhibitors. In a study of the skin-aging-related activities of *Rosa* spp., flavonoid content extracted from *R. gallica* petals was found to significantly suppress tyrosinase activity, melanin production, and solar-UV-induced matrix metalloproteinase [82]. A polyphenol from *R. rugosa* flower tea increases the mean lifespan and enhances thermotolerance and resistance to oxidative stress in *C. elegans*, owing to its powerful antioxidant effects in vitro and strong protection against oxidative DNA damage [81]. Recently, studies performed in vitro and in vivo, as well as clinical trials, have investigated the skin-whitening and anti-wrinkle activities of *R. gallica* L. petal extracts [83]. In an in vitro study, RPE reduced melanin accumulation in human B16F10 melanoma cells and exhibited anti-wrinkle activity in human dermal fibroblasts. In an in vivo study, RPE inhibited solar-ultraviolet-stimulated MMP-1 expression via c-Jun regulation. In a clinical trial, volunteers who underwent facial skin RPE treatment exhibited significant skin brightness. *R. roxburghii* could significantly prevent the skin aging in D-galactose-induced mice by enhancing the activity of SOD, reducing the accumulation of MDA [41]. To evaluate moisture-preserving activities, the moisture absorption and moisture retention activities of polysaccharides from *R. rugosa* petals were tested in vitro by controlling the relative humidity of the desiccators, and they were found to exhibit strong moisture-preserving activity [49]. Extracts from *R. rugosa* were able to alleviate excessive androgenic-induced acne in a golden hamster acne model [84], which may also affect the regulation of sex hormone levels.

## 4. Cascade Utilization of *Rosa* spp.

**Figure 2:**
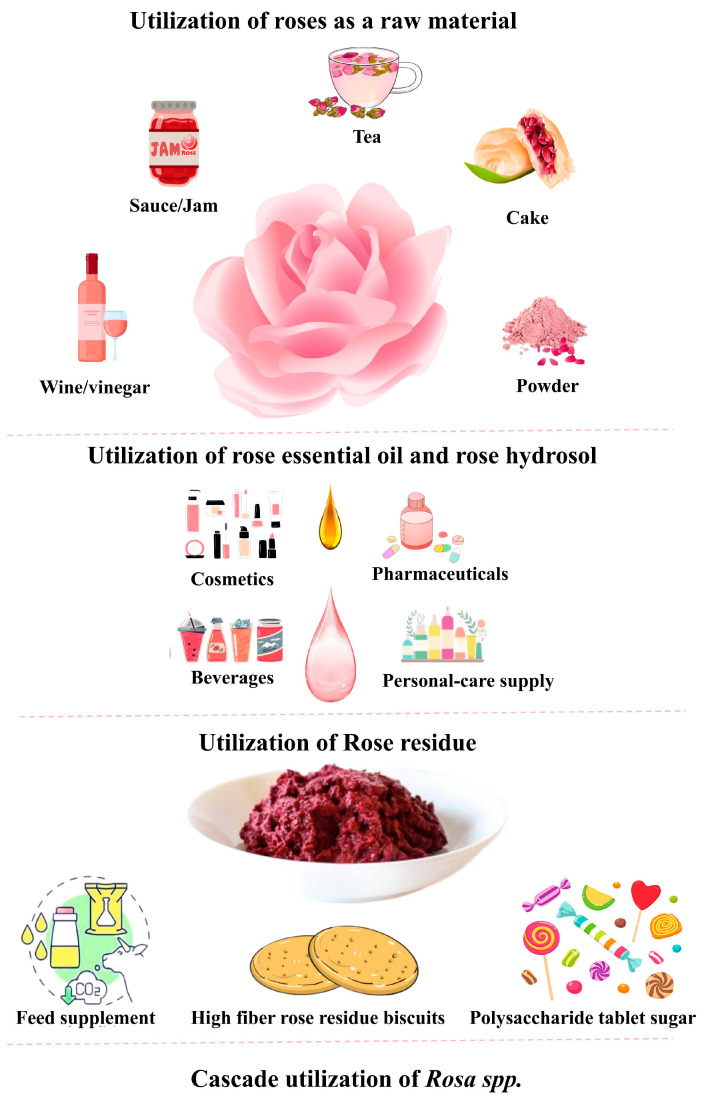
Cascade utilization of *Rosa* spp.

Roses have medicinal, edible, and ornamental value and are increasingly loved by people. Since the raw materials of roses, rose essential oil, hydrosol, and the remaining rose residues contain various bioactive components, a cascading utilization approach has been proposed to maximize the application of this resource (Figure 2).

### 4.1. Utilization of Raw Materials

The raw materials of roses are widely used in the production of various foods and beverages, such as tea, sauces, jam, wine, vinegar, and cake.

Owing to their richness in alcohols, terpenes, and various volatile aromatic components, rose flowers yield a tea with a pleasant fragrance. Regular consumption of rose flower tea is associated with the benefits of clearing heat, detoxification, promoting metabolism, nourishing the liver and stomach, and regulating hormones [86]. An analysis of rose flower tea and methanol extracts revealed significantly higher copper content in rose flower tea than in methanol extracts, accompanied by notable antioxidant effects [81].

Rose flower sauce, which contains volatile compounds such as aldehydes, alcohols, and esters, boasts a natural rose aroma that is widely appreciated for its taste and flavor [87]. Rose flowers are rich in nutrients and novel methods have been adopted to preserve these nutrients during processing. For instance, using fresh flower petals as the base material and adding ingredients such as white sugar, citric acid, pectin, and salt results in a new type of rose sauce [88]. This approach offers a simple processing technique that enhances the color, aroma, taste, and appearance of the product compared with traditional methods. It not only maintains nutritional value but also elevates flavor. Sweet products produced using rose flower sauce, such as mooncakes, bread, and pastries, enhance the color and fragrance of food and provide a unique flavor [89].

Rose vinegar is naturally fermented with plant materials, resulting in a natural rose-red color, mild flavor, and strong vinegar aroma. This has made it popular among the public. It is possible to increase aroma components and vinegar content by improving the processing techniques and fermentation methods [90]. Additionally, these modifications can positively affect the taste, color, nutritional value, and production efficiency of vinegar [91].

Rose wine, which has deep historical and cultural significance, features a rich and aromatic flavor characterized by its sweetness and aftertaste. Current research on the craftsmanship of rose wine has matured, resulting in products not only rich in rose aroma but also imbued with amino acids and phenolic substances from the fermentation process [92]. It imparts an appealing aroma and provides both nutritional and health benefits [93].

Roses are also used in a variety of desserts, pastries, and snacks and are valued for their numerous beneficial components. This has led to their extensive use in various forms, including dishes such as rock candy rose tea [94], which provides a sweet and fragrant experience while helping to disperse blood stasis. Rose porridge is another popular health food that promotes the dispersion of blood stasis. Rose flower buds combined with brown sugar can be made into a paste for consumption to support blood nourishment and skin rejuvenation.

### 4.2. Utilization of Rose Essential Oil and Rose Hydrosol

Rose essential oil is a precious natural fragrance that serves as a vital ingredient in the production of high-grade cosmetics, perfumes, and food products, and it ranks among elite flower essential oils [95]. The oil exhibits properties such as promoting digestion, improving blood circulation, and relieving pain; it has potential benefits for peripheral and visceral microcirculation [96].

The diverse chemical composition of rose essential oils, including terpenes, alcohols, esters, ethers, aldehydes, and alkanes, contributes to cell regeneration, enhances blood circulation, and provides soothing and sedative effects to aid sleep [97]. In addition, it possesses antibacterial, anti-thrombotic, and antioxidant properties. Its high antimicrobial activity, along with its substantial content of tocopherols and carotenoids, makes rose essential oil a natural preservative in the food industry, capable of reducing costs and mitigating the risks associated with the extensive use of artificial preservatives [5].

Rose hydrosol, a by-product of rose essential oil distillation, is primarily composed of compounds such as citronellol, nerol, and eugenol, imparting a rich and fragrant aroma [98]. It is notable for its pronounced efficacy in alleviating stress and is suitable as a component of composite preservatives, demonstrating favorable application effects. Research has demonstrated the potent inhibitory effects of rose hydrosol on bacteria [99], whereas its effects on molds and yeasts are less significant.

### 4.3. Applications of High-Value Compounds Extracted from Rosa spp. By-Products

Rose residues are the waste produced during the extraction of rose essential oils. The main products of rose processing are rose essential oil and rose hydrosol, of which 80% of the by-products are rose residues. The extraction of rose essential oil only extracts volatile components, such as rose aroma, whereas other non-volatile components (such as proteins and minerals) remain in the residue, with prominent nutrients and high reuse value.

Rose residues are mostly discarded as garbage, wasting resources and polluting the environment. Studies have shown that rose residues are rich in nutrients [100], with a relatively high content, and that they contain essential amino acids for humans. Additionally, they exhibit elevated levels of crude fat and crude fiber and a substantial amount of vitamin E. The mineral element profiles are also diverse. The analysis of rose petal residues reveals significant nutritional components. They include crude fat, crude fiber, vitamin E, calcium, zinc, iron, copper, magnesium, manganese, and phosphorus. This nutritional information highlights the potential benefits of using rose petal residues in animal diets and their positive impact on health due to their rich content of various essential nutrients and minerals.

Rose residue is a natural raw material that can be further developed and utilized. Currently, the extraction, separation, and determination of the active compounds in rose residues primarily focus on polyphenols, polysaccharides, and flavonoids. Studies on the efficacy of rose residues have primarily focused on their antioxidant activity, with most studies conducted at the national level rather than internationally. Thus, rose residues are a natural resource that is worthy of further exploration.

Rose residues can also be used to develop products such as rose petal jams by incorporating rose petals into pomace. Studies have shown that they can be used to produce cookies, candies, tea, and animal feed additives. This allows for the transformation of pomace from waste to a valuable resource, and the reutilization of rose residue for the production of rose essential oil. Such practices not only yield economic benefits but also address waste management concerns. The rational and comprehensive utilization of large quantities of rose pomace has emerged as a pressing challenge in essential oil production for roses.

Traditional waste handling methods incur costs related to disposal and pollution mitigation. However, advanced valorization pathways can transform waste into high-value products, improving profitability. Technologies for extracting bioactive compounds from rose waste, developing functional foods, and producing animal feed additives have demonstrated success [14]. Additionally, bioremediation approaches using algal–fungal systems degrade organic pollutants in wastewater, achieving COD removal and ammonia nitrogen reduction [101]. These processes reduce environmental impact while generating revenue streams.

Cascading utilization technologies we mentioned in our review can increase the value of rose products. Despite progress, gaps remain in global waste volume data and detailed cost–benefit analyses, underscoring the need for further research. Embracing circular economy principles in the rose industry not only mitigates waste but also unlocks new markets for nutraceuticals, cosmetics, and functional materials, aligning sustainability with economic growth.

## 5. Conclusions

The COVID-19 pandemic has increased people’s awareness of healthcare. Roses have attracted increasing attention due to their diverse biological functions as well as their dual medicinal and edible properties; however, the medicinal and food-related effects of roses have not yet been fully explored. Moreover, there remains a lack of systematic explorations of the pharmacological research and development process for studying the medicinal properties of roses and their by-products. Further research is needed to explore processed products and improve the related processing technologies.

## Figures and Tables

**Table 1:** The bioactive components and potential mechanisms of *Rosa* in vivo and in clinical trials.

Constituent	Study Type	Animal Model/Subjects	Dosages	Potential Mechanisms	Ref.
**Antioxidant effect**					
*R. rugosa* extract	In vivo	Nrf2-knockout zebrafish	600–1000 µg/mL	Enhanced oxidative stress resistance; upregulated the antioxidant genes	[21]
*R. damascena*	clinical trial	high-risk pregnant women	250 mL	The intervention MDA decreased by 1.87 ± 0.45 μmol/L, the control reduction was 0.42 ± 0.31 μmol/L	[22]
**Anti-inflammatory activity**					
50% ethanol extract *R. rugosa*	In vivo	1-chloro-2,4-dinitrobenzene-induced ear edema mouse model	50, 100, and 200 mg/kg	Decreased the elevated levels of IgE/IgG; suppressed pro-inflammatory cytokines and chemokines in ear tissues	[23]
*R. laevigata* extract	In vivo	BALB/c mice	50 and 100 mg/kg	Attenuated allergic airway inflammation by reducing inflammatory cells, the secretion of IgE and related cytokines	[24]
*R. roxburghii* flavonoids	In vivo	Male Kunming mice	30 and 60 mg/kg	Inhibited the radiation-induced apoptosis by reducing the cleavage of these caspases in a dose-dependent manner	[25]
Rosebud extract		Male Sprague Dawley rats	10, 30, and 100 mg/kg	Decreased λ-carrageenan-induced tissue exudation, inflammatory cytokines and cell infiltration; inhibited prostaglandin E2	[26]
**Antibacterial activity**					
Rosehip extract -functionalized nanoparticles	In vivo	*Galleria mellonella* invertebrate animal model	225 mg/kg	Disrupted cellular structures (bacterial cell wall and cytoplasmic membrane) and damaged the bacterial cell	[27]
**Anti-diabetic activity**					
*R. roxburghii* fruit polysaccharide	In vivo	Male obese diabetic db/db mice	300, 600, and 900 mg/kg/d for 8 weeks	Attenuated hyperlipidemia by regulating the gene expression of lipid metabolism; reversed gut dysbiosis; enhanced beneficial bacteria abundances	[28]
*R. rugosa* polyphenol -enriched extract	In vivo	Male rat dyslipidemia model (high-fat diet + STZ injection)	37.5, 75, and 150 mg/kg for 4 weeks	Improved hepatic steatosis and liver function via the induction of AMPK signaling activity	[29]
*R. canina* fruits oligosaccharide	In vivo	Male STZ-induced diabetic Wistar rats	8–40 mg/kg for 3 weeks	Improved pancreatic β-cells and tissue pathological changes by increasing the expression of Ngn3, Nkx6.1, and insulin	[30]
**Anti-cancer activity**					
*R. laevigata* fruits polysaccharide	In vivo	Zebrafish model	100, 200, and 400 μg/mL	Inhibited HepG2 cell migration by regulating the FAK signaling pathway	[31]
*R. rugosa* herbal combinatory	In vivo	A breast cancer rat model	1.25, 2.5, 5, 10, and 20 mg/mL	Down-regulated the level of serum estradiol and suppressed the protein expression in the SNCG/ER-alpha/AKT-ERK pathway	[32]
**Neuroprotection**					
*R. damascena* extract	In vivo	Aluminum chloride-induced Alzheimer’s model of Wister rats	500 and 1000 mg/kg	Increased the levels of catalase and glutathione; attenuated MDA levels; regulated AChE activity	[33]
*R. damascena* oil	clinical trial	Randomized clinical trial of nulliparous women	0.08 mL essence	Reduced the severity of pain and anxiety in the first stage of labor	[34]
*R. damascena* oil	clinical trial	Patients underwent septorhinoplasty/rhinoplasty surgery	0.2 mL	Reduced preoperative anxiety of patients undergoing septorhinoplasty/rhinoplasty	[35]
**Gastrointestinal protection**					
*R. laevigata* polysaccharides	In vivo	Sodium dextran sulfate-induced beagles	400 mg/kg	Alleviated colitis by preserving the intestinal barrier and regulating the gut microbiota composition	[36]
*R. roxburghii* fruit polysaccharide	In vivo	Male C57BL/6J mice colitis model induced by high-fat diet	400 mg/kg	Ameliorated HFD-induced colitis in mice by modulating gut microbiota	[37]
**Hepatorenal protection**					
*R. roxburghii* fruit freeze-dried powder	In vivo	Rat model of unilateral ureteral obstruction	3 and 6 g/kg	Prevented renal fibrosis and impairment, associated with the inhibition of oxidative stress and TGF-β1/Smads signaling	[38]
*R. laevigata* polysaccharide	In vivo	Diabetic nephropathy mouse model	40 and 80 mg/kg	Modulated tryptophan metabolism and inhibited ferroptosis and PI3K/AKT pathway-mediated apoptosis in the kidney	[39]
*R. laevigata* total flavonoids	In vivo	LPS-induced liver injury mice	50, 100, and 200 mg/kg	Exhibited liver-protective effects by altering FXR-mediated oxidative stress, inflammation, and lipid metabolism	[40]
**Skin protection**					
*R. roxburghii* extract	In vivo	D-galactose-induced mice skin aging	0.5, 1, and 2 g/kg	Enhanced the activity of SOD in skin tissue, reduced the accumulation of MDA; increased the level of HYP and HA	[41]
*R. rugosa* polysaccharide	In vivo	Imiquimod-induced psoriasis mouse model	10, 50, and 100 mg/kg	Suppressed the PI3K-AKT/mTOR pathway	[42]

## Data Availability

No new data were created or analyzed in this study. Data sharing is not applicable to this article.
